# Improved Thermoelectric Properties and Environmental Stability of Conducting PEDOT:PSS Films Post-treated With Imidazolium Ionic Liquids

**DOI:** 10.3389/fchem.2019.00870

**Published:** 2020-01-08

**Authors:** Temesgen Atnafu Yemata, Yun Zheng, Aung Ko Ko Kyaw, Xizu Wang, Jing Song, Wee Shong Chin, Jianwei Xu

**Affiliations:** ^1^Institute of Materials Research and Engineering, Agency for Science, Technology, and Research (A^*^STAR), Singapore, Singapore; ^2^Department of Chemistry, National University of Singapore, Singapore, Singapore; ^3^Department of Electrical and Electronic Engineering, Southern University of Science and Technology, Shenzhen, China

**Keywords:** conducting polymer, PEDOT:PSS, thermoelectric, ionic liquids, environmental stability

## Abstract

Poly(3,4-ethylenedioxythiophene):poly(styrenesulfonate) (PEDOT:PSS) is one of the most popular conducting polymers and widely used as polymer thermoelectric materials, and its thermoelectric performance could be improved by a variety of post-treatment processes. This paper reported two series of post-treatment methods to enhance the thermoelectric performance. The first series method included pre-treatment of PEDOT:PSS film with formamide, followed by imidazolium-based ionic liquids. The second series method included pre-treatment of PEDOT:PSS film with formamide, followed by sodium formaldehyde sulfoxylate, and finally imidazolium-based ionic liquids. Two series of post-treatment methods significantly improved the power factor of PEDOT:PSS when compared to that of PEDOT:PSS treated with formamide only. For example, using the first series post-treatment method with 40 vol.% ionic liquid 1-butyl-3-methylimidazolium bis(trifluoromethanesulfonyl) amide, the Seebeck coefficient of the PEDOT:PSS film increased from 14.9 to 28.5 μV/K although the electrical conductivity reduced from 2,873 to 1,701 S/cm, resulting in a substantial improvement in the overall power factor from 63.6 to 137.8 μW/K^2^m. The electrical conductivity enhancement in the formamide-treatment process was in part ascribed to the removal of the insulating PSS component. Further treatment of PEDOT:PSS film with ionic liquid caused dedoping of PEDOT and hence increased in Seebeck coefficient. In contrast, second series post-treatment method led to the reduction in electrical conductivity from 2,873 to 641 S/cm but a big improvement in the Seebeck coefficient from 14.9 to 61.1 μV/K and thus the overall power factor reached up to ~239.2 μW/K^2^m. Apart from the improvement in electrical conductivity, the increase in Seebeck coefficient is on account of the substantial dedoping of PEDOT polymer to its neutral form and thus leads to the big improvement of its Seebeck coefficient. The environmental stability of ionic liquid-treated PEDOT:PSS films were examined. It was found that the ionic liquid treated PEDOT:PSS retained more than 70% Seebeck coefficient and electrical conductivity at 75% RH humidity and 70°C for 480 h. The improved long-term TE stability is attributed to the strong ionic interaction between sulfonate anions and bulky imidazolium cations that effectively block the penetration of water and lessen the tendency to take up water from the air.

## Introduction

Thermoelectric (TE) materials are able to directly convert heat into electricity and *vice versa* (Bell, 2008; Snyder and Toberer, 2011). TE devices have been thought as promising “green” power generators and they play an essential role in harvesting low-grade heat like waste heat or exhausted heat that is usually dissipated to the environment. The performance of TE materials largely depends on the dimensionless figure-of-merit (*ZT*), *ZT* = σ*S*^2^*T*/κ where σ is electrical conductivity, *S* is Seebeck coefficient, *T* is absolute temperature, and κ is thermal conductivity (Snyder and Toberer, 2011). More recently, traditional inorganic materials such as SnSe (Chang et al., 2018; Lee et al., 2019), PbTe (Tan et al., 2016; Chen et al., 2017), GeTe (Li et al., 2018), and Cu_2_Se_0.5_S_0.5_ (Ren, 2017) with *ZT* values of over 2 have been reported. Despite their impressive TE performance, the drawbacks such as high cost, scarcity, toxicity, and low processability limit their commercial applications. Moreover, most of these promising inorganic TE materials have good performance when operating temperatures exceed 300–400°C. They may not function well for ambient temperature heat recovery as a large amount of waste heat in our surroundings is below 200°C (Yoo et al., 2015). Therefore, organic TE materials with high performance at ambient temperature have gained increasing interest in recovering a huge amount of low temperature waste heat (Yoo et al., 2015).

Conductive polymers (CPs) have immerged as promising TE materials due to the tunable σ, low κ, and relatively low production cost. The commonly studied CPs include polyaniline (PANi) (Yoon et al., 1995; Mateeva et al., 1998; MacDiarmid, 2001), poly(3,4-ethylenedioxythiophene):poly(styrenesulfonate) (PEDOT:PSS) (Zhang et al., 2010), polypyrrole (PPy) (Kemp et al., 1999), polyacetylene (PAc) (Kaneko et al., 1993), and polythiophene (PTH) (MacDiarmid, 2001; Hu et al., 2013). Other CPs, like uniaxially aligned iodine (I_2_)-doped PAc, has a power factor (*PF* = *S*^2^σ) of ~ 1,350 μW/K^2^m, but it has no practical application due to its poor stability and processability (Cowen et al., 2017). On the contrary, PPy and PANi have good stability but poor *PFs* of less than 10 μW/K^2^m (Li et al., 2010; Liang et al., 2017). Currently, the TE performance of CPs cannot rival that of state-of-the-art inorganic counterparts. However, the TE performance of CPs has been improved significantly over the years, showing very promising *ZT* values for applications.

Recently, among the CPs, PEDOT:PSS has attracted great attention due to the high σ, intrinsically low κ, water-processability, and commercial availability (Khan et al., 2015; Wei et al., 2015). Several approaches have been widely investigated to enhance the TE performance of PEDOT:PSS, such as post treatment (Fan et al., 2017), electrochemical oxidation (Park et al., 2013), and hybrid approach (Zhang et al., 2010), etc. PEDOT:PSS can form hybrids with different carbon sources, metallic nanomaterials or inorganic TE materials. This allows hybrid materials to tap on the advantages of each component, such as a large *S* or σ to balance individual TE characteristics to give an optimum TE performance (Zhang et al., 2010; Moriarty et al., 2013; Xu et al., 2013; Park et al., 2014a). The other challenge is the incompatibility of the two components (i.e., PEDOT:PSS and additives) as observed in many cases, where phase separation of a single component leads to non-uniform films. Surface modification through post-treatment (Mcgrail et al., 2015; Wei et al., 2015) has also proven effective in improving the TE performance of PEDOT:PSS films by removing the insulating PSS segment from PEDOT:PSS. The typical process is to immerse PEDOT:PSS film in dimethyl sulfoxide (DMSO), ethylene glycol (EG) (Culebras et al., 2014; Park et al., 2014b) or inorganic acid solution. Inorganic salts and organic solvents with high dielectric constants have demonstrated the ability in increasing the σ of PEDOT by a few orders of magnitude, giving rise to a significant enhancement in *PF* (Zhang et al., 2010; Culebras et al., 2014). The highest *PF* can reach up to 469 μW/K^2^m, leading to a large *ZT* value of 0.42 at 300 K (Kim et al., 2013). These post-treatments can increase the concentration of charge carriers and bipolarons. However, the high doping level in general results in a small *S* because of the extra charge carriers (Park et al., 2014b). Therefore, appropriate methods that improve *S* are desired as the *ZT* involves the square of *S*. Previous studies already demonstrated that the *PF* could be improved by regulating the redox level through an electrochemical or chemical method (Tsai et al., 2011; Bubnova et al., 2012), thus achieving the optimum TE properties through the control of charge carrier concentration. For instance, Park et al. reported PEDOT:PSS film with an optimized *PF* of 112 μW/K^2^m by treating with a mixture of DMSO and hydrazine (Park et al., 2014b). Also, Lee et al. employed a multistep process of ultrafiltration and dedoping by hydrazine to treat PEDOT:PSS and achieved a *PF* of 115.5 μW/K^2^m (Lee et al., 2014a). Moreover, Park et al. reported an improved *PF* of 1,270 μW/K^2^m by controlling the electrochemical oxidation of PEDOT films (Park et al., 2013). Recently, Fan et al. treated PEDOT:PSS films with sulfuric acid and different concentrations of sodium hydroxide. They reported an improved σ of 2,170 S/cm, an *S* of 39.2 μV/K, and hence a *PF* of 334 μW/K^2^m at room temperature (Fan et al., 2017). More recently, Fan et al. have demonstrated that 1-ethyl-3-methylimidazolium dicyanamide (EMIM-DCA) treated PEDOT:PSS films had an ultrahigh *PF* of 754 μW/K^2^m and a ZT of 0.75 at room temperature (Fan et al., 2018). Moreover, Saxena et al. treated PEDOT:PSS film with EMIM-DCA in THF solution. They observed the simultaneous improvement of σ and *S*, and a maximum *PF* of 170 μW/K^2^m had been obtained because of the binary nature of both ionic liquids and PEDOT:PSS (Saxena et al., 2019). Therefore, approaches that can only slightly lower the high σ of formamide-treated PEDOT:PSS films while significantly improve the *S* could be effective for improving the TE properties of PEDOT:PSS films.

In this work, we reported the enhancement of the *S* and *ZT* of PEDOT:PSS films with ionic liquids (ILs) treatment. The effect of anions associated with ILs on the TE properties of treated PEDOT:PSS films were also investigated, revealing that the type of anions played a somewhat role in affecting the TE properties. PEDOT:PSS films treated with 40 vol.% IL, 1-butyl-3-methylimidazolium bis(trifluoromethanesulfonyl) amide (BMIM-TFSI), achieved a highest *PF* of 239.2 μW/K^2^m, and it also demonstrated a very good environmental stability, indicating that our approach is potential for practical TE application in the future.

## Experimental Section

### Materials

**Scheme 1:**
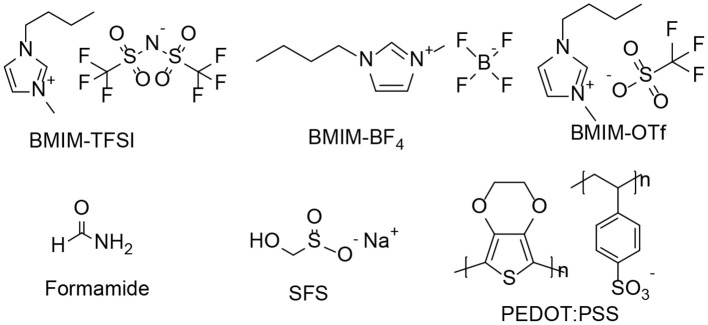
Chemical structures of BMIM-TFSI, BMIM-BF_4_, BMIM-OTF, SFS, formamide, and PEDOT:PSS.

PEDOT:PSS solution (Clevios PH 1000, PEDOT:PSS weight ratio = 1:2.5 and concentration by mass = 1.3%) was purchased from Heraeus. Formamide, sodium formaldehyde sulfoxylate (SFS), 1-butyl-3-methylimidazolium tetrafluoroborate (BMIM-BF4), 1-butyl-3-methylimidazolium trifluoromethanesulfonate (BMIM-OTf) or 1-butyl-3-methylimidazolium bis(trifluoromethanesulfonyl) amide (BMIM-TFSI) were purchased from Sigma-Aldrich. All chemicals were used as received without further purification. The chemical structures of ionic liquids are shown in Scheme 1.

### Sample Preparation

PEDOT:PSS solution was filtered using a 0.45 μm poly (vinylene difluoride) PVDF syringe filter. The glass substrates were cleaned with deionized (DI) water, detergent, acetone, and isopropanol in an ultrasonic bath consecutively and dried with nitrogen gas. The glass substrate was subjected to ultraviolet (UV)-ozone surface treatment for 15 min before use.

#### Pristine PEDOT:PSS Films

PEDOT:PSS films were prepared by drop-casting a 300 μL of PEDOT:PSS solution on the pre-cleaned glass substrate using a micropipette. The deposited sample was firstly dried in air at 50°C for 30 min to mainly drive off the H_2_O solvent and then kept at 80°C for another 10 min to further remove the residual solvent. Finally, the PEDOT:PSS films were annealed at 130°C for 10 min to ensure complete evaporation of the solvent. The final thickness of each dried PEDOT:PSS film was in the range of 8–10 μm.

#### Formamide Post-treatment

For the post-treatment process, 140 μL formamide was first dropped onto the PEDOT:PSS films on a hot plate at 180°C and then the films were dried for about 10 min to remove the residual formamide. Afterward, the formamide treated PEDOT:PSS films were cooled to room temperature in air, and these films were rinsed with DI water and then dried again on a hot plate at 140°C for 5 min. The detailed procedures for treatment can be found in our previous work (Kyaw et al., 2018).

#### Sodium Formaldehyde Sulfoxylate (SFS) Post-treatment

Next, treatment with a salt solution was performed by dropping 150 μL aqueous SFS solution onto a formamide pretreated PEDOT:PSS film on a hot plate at 140°C. The film became dry after ~5 min. The SFS treated PEDOT:PSS film was cooled to room temperature in air, rinsed with DI water for three times to wash away the salt, and then dried at 140°C again.

#### IL Post-treatment

**Scheme 2:**
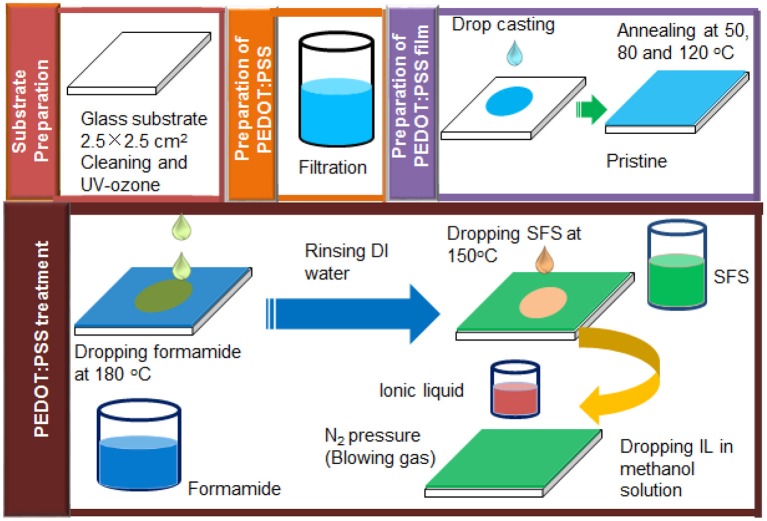
Schematic of sample preparation for pristine and various IL-treated PEDOT:PSS films.

The post-treatment with ILs was conducted at room temperature. Initially, three ILs (ILs) (BMIM-TFSI, BMIM-BF_4_, and BMIM-OTf) with concentrations of i.e., 0, 20, 40, 60, 80, and 100 vol.% ILs in methanol were prepared. Then pre-treated PEDOT:PSS films with formamide and SFS were further treated with ILs in methanol according to the following steps: 150 μl IL in methanol was dropped onto PEDOT:PSS film at room temperature and left for 30 min. Next, the samples were dried by the aid of blowing N_2_ gas at a pressure of 0.15 MPa for another 30 min to remove the residual solvent of IL. Then the films were rinsed by dipping the films in DI water for three times and then finally annealed at an elevated temperature of 140°C under air to remove the residual solvent. These treated samples were cooled down to room temperature before TE property measurements. All treatments were conducted at their optimized treatment temperatures and conditions. Scheme 2 illustrates detailed PEDOT:PSS film preparation and post-treatment with formamide, SFS, and various ILs.

### Characterization

The thicknesses of PEDOT:PSS films was measured before and after treatment using KLA-Tencor P-10 surface profiler with a detection limit of 100 Å (10 nm). The sheet resistance (*Rs*) of the films was determined by the four-point probe method (Laresta-GP MCP-T610 from Mitsubishi Chemical) at the room temperature. The Loresta-GP MCP-T610 meter includes standard accessories PSP probe (MCP-TP06P [4-pins, inter-pin distance 1.5 mm, pin points 0.26R, spring pressure 70 g/pin is intelligent)] and probe checker [RMH112 (MCP-TP06P)]. The edges of the film were located at 10 mm from the measurement point. The σ is the inverse of the resistivity ρ_*H*_, which is calculated in terms of the equation: ρ_*H*_ = *R*_*S*_*t* where *Rs* and *t* are resistance and thickness of the film, respectively (Krupka, 2013).

The *S* was obtained with a homemade setup in a humidity-controlled room with a relative humidity (RH) of 55% (Figure S1). It consists of two stages (about 5 mm apart), one of which is integrated with a heater to generate a temperature gradient in the test sample. First, two Au electrodes with 20 mm long, 1 mm wide, and 2 mm apart were thermally evaporated on the film. To minimize the experimental error, PEDOT: PSS film outside the area of electrodes was removed. The Au electrodes were connected to a Keithley 2,400 source meter through the probes to obtain the voltage difference (Δ*V*). Simultaneously, K-type thermocouples were connected to a data logger (Omron ZR-RX45) to collect the actual temperatures of the PEDOT:PSS film. The voltage probes and thermocouples were placed at the same temperature zone on each side so that the measured voltage corresponded to the actual thermal gradient between the two voltage probes. The measured thermovoltage was corrected by the thermovoltage of Au wire to obtain the absolute *S* of the films. *S* was estimated based on the slope of the linear relationship between thermoelectric voltage and the temperature difference of the two probes (i.e., *S* = −Δ*V*/Δ*T*). The Hall coefficient *R*_H_ was measured using a Hall-effect measurement system (Ecopia HMS-5000) with the van der Pauw method. First, the Ag electrodes were deposited onto the film through a shadow mask. The carrier concentration *n* and mobility μ were calculated using the following equations: *n* = *1/ (|R*_*H*_*|*× *e) and* μ = *|R*_*H*_*|/*ρ_*H*_, where *e* is electron charge. The κ was calculated using the equation κ = *b*^2^ */Cp*ρ_*m*_, where ρ_*m*_, *b, Cp* are density, thermal effusivity, and specific heat capacity, respectively. The *b* was measured with a Pulsed Light Heating Nano TR (NETZSCH) system with an ultrafast pulsed laser flash method using the front heating-front detection mode, which is designed for the thermal analysis of 30 nm −20 μm thin films. The *Cp* was measured independently by differential scanning calorimeter (DSC) (Mettlier Toledo DSC1). The ρ_*m*_ at room temperature was calculated from the mass and volume of the film. For all measurements, at least 10 samples for each measurement were prepared at the same conditions, and measurements were carried out at least five times for each sample to obtain the statistical results. X-ray diffraction (XRD) patterns of the films were obtained by a D8 Advance System (Bruker Corporation) equipped with a Cu *K*_α_ X-ray source, λ = 0.15406 nm. The Raman spectroscopy measurements were conducted on a Raman microscope (Renishaw) with a laser wavelength of 785.5 nm, a laser beam spot size of 200 μm and an accumulation time of 30 s. The absorption spectra measurement was performed on a UV-Vis-NIR spectrophotometer (Shimadzu, UV-3600). The films were spin-coated on quartz substrates. X-ray photoelectron spectroscopy (XPS) of the films were obtained by the Theta Probe Angle-Resolved X-ray Photoelectron Spectrometer (ARXPS) System (Thermo Scientific) using monochromated, micro-focused Al *K*_α_ X-ray photons (*h*υ = 1486.6 *eV*) at a base pressure of 1 × 10^−9^ Torr and a step size of 0.1 eV. The curve fitting was carried out using the Avantage software. Atomic force microscopy (AFM) images were taken on a Bruker Dimension Icon AFM using the tapping mode. Ultraviolet photoelectron spectroscopy (UPS) measurement of the films was obtained by using the He I photon (21.22 eV) radiation line from a discharge lamp, with an experimental resolution of 0.15 eV. All UPS measurements of the onset of photoemission for determining the Φ were performed using standard procedures with a −4.5 V bias applied to the sample. The films were prepared by drop-casting a PEDOT:PSS solution onto a pre-cleaned silicon substrate.

### Stability Study of PEDOT:PSS Films

PEDOT:PSS films were placed in a humidity controlled chamber and their TE performance was measured at varied temperature and humidity conditions in order to study the effect of humidity on TE properties of PEDOT:PSS films (Kim et al., 2016). In this study, the stability study was conducted using a constant climate chamber (Memmert HPP 110) in the temperature range from +0°C to +70°C, as well as the active humidification and dehumidification from 10 to 90% RH.

## Results and Discussion

### Film Post-treatment and TE Properties

Three different ILs, 1-butyl-3-methylimidazolium bis(trifluoromethanesulfonyl) amide (BMIM-TFSI), 1-butyl-3-methylimidazolium tetrafluoroborate (BMIM-BF_4_) or 1-butyl-3-methylimidazolium trifluoromethanesulfonate (BMIM-OTf) in methanol were used for this study. ILs are composed of positively and negatively charged species (i.e., binary nature). In these three ILs, the cation is always BMIM, and only the anion is different. All post-treatment methods are summarized in Table 1.

**Table 1:** Post-treatments methods of PEDOT:PSS films.

**Post-treatment Series**	**Post-treatment methods**	**Reagents used**	**Procedures**
1	F-PEDOT:PSS	Formamide	Drop 140 μL formamide onto the PEDOT:PSS films at 180°C and dry for about 10 min. Then rinse with DI water and dry again on a hot plate at 140°C for 5 min.
	ILs-F-PEDOT:PSS	Formamide + Ionic liquid	The three ILs (BMIM-TFSI, BMIM-BF4, and BMIM-OTf) with 0, 20, 40, 60, 80, and 100 vol.% ILs in methanol were prepared and was dropped onto F-PEDOT:PSS film at room temperature and left for 30 min and finally dry blowing N_2_ gas. Then rinse with DI water and dry again on a hot plate at 140°C for 5 min.
2	F-PEDOT:PSS	Formamide	Drop 140 μL formamide onto the PEDOT:PSS films at 180°C and dry for about 10 min. Then rinse with DI water and dry again on a hot plate at 140°C for 5 min.
	SFS-F-PEDOT:PSS	Formamide + Sodium formaldehyde sulfoxylate	Drop 150 μL aqueous SFS solution onto F-PEDOT:PSS film at 140°C and dry for about 5 min. Then rinse with DI water and dry again on a hot plate at 140°C for 5 min.
	ILs-SFS-F-PEDOT:PSS	Formamide + Sodium formaldehyde sulfoxylate + Ionic liquid	The three ILs (BMIM-TFSI, BMIM-BF4, and BMIM-OTf) with 0, 20, 40, 60, 80, and 100 vol.% ILs in methanol were prepared and was dropped onto SFS-F-PEDOT:PSS film at room temperature and left for 30 min and finally dry blowing N_2_ gas. Then rinse with DI water and dry again on a hot plate at 140°C for 5 min.

**Figure 1:**
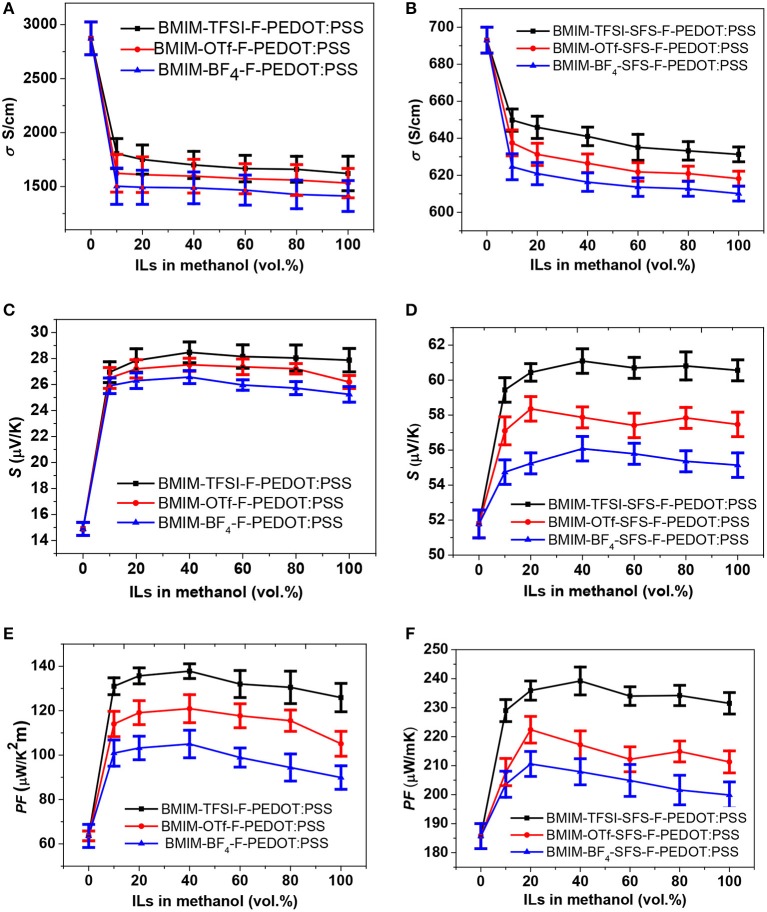
TE properties of PEDOT:PSS films vs. vol.% ILs. ILs-F-PEDOT:PSS films: **(A)** σ, **(C)** *S*, and **(E)** *PF*; ILs-SFS-F-PEDOT:PSS films: **(B)** σ, **(D)** *S*, and **(F)** *PF*.

Figures 1A,C,E show the *S*, σ, and *PF* of the PEDOT:PSS films treated by sequential formamide and ILs with various concentrations. The PEDOT:PSS films were pre-treated with formamide, and then ILs in methanol were dispersed onto the PEDOT:PSS films (first series post-treatment: IL-F-PEDOT:PSS). Introduction of IL treatment reduced the σ, while noticeably improved the *S* of IL-F-PEDOT:PSS films. It is worth noting that the σ and *S* of IL-F-PEDOT:PSS films almost remain unchanged when the concentration of ILs increases from 20 to 80%, suggesting that they are less affected by the concentration of the ILs. Treatment with 20–100 vol.% BMIM-TFSI (i.e., BMIM-TFSI-F-PEDOT:PSS) led to an enhancement of the *S* from ~14.9 to ~28.1 μV/K and a significant reduction in the σ from ~2,873 to ~1,678 S/cm. Overall, in comparison to the *PF* of around 65 μW/K^2^m without IL treatment, the optimal *PF* reaches 137.8 μW/K^2^m when 40 vol.% BMIM-TFSI solution is used. Interestingly, the type of anions plays a certain role in improving *S*. TFSI is the most efficient to enhance the magnitude of the *S*, followed by OTf and BF_4_. The possible rationale is because of the binary nature of both PEDOT:PSS and ILs, which consist of positively and negatively charged species. These charged species in ILs, for example, negatively charged TFSI and positively charged BMIM, will interact with correspondingly positively charged polymer PEDOT and negatively charged PSS chains via electrostatic interactions.

In the second series post-treatment, SFS was introduced to treat PEDOT:PSS before applying ILs treatment. Based on our previous findings (Yemata et al., unpublished), subsequent treatment of F-PEDOT:PSS with SFS can reduce the doping level, and as a result, improve the *PF* of PEDOT:PSS films. The σ, *S*, and *PF* of the ILs-SFS-F-PEDOT:PSS films with different concentrations of ILs are summarized in Figures 1B,D,F. The σ of ILs-SFS-F-PEDOT:PSS film was only slightly lower than that of the SFS-F-PEDOT:PSS films (631–649 vs. 693 S/cm), while the S became higher. BMIM-TFSI-SFS-F-PEDOT:PSS films exhibited an improved *S* of ~60.8 μV/K compared to SFS-F-PEDOT:PSS (51.8 μV/K), while the σ (~631 S/cm) decreased slightly. Consequently, the *PF* of BMIM-TFSI-SFS-F-PEDOT:PSS reached 239 μW/K^2^m, about ~29% enhancement over that of F-SFS-PEDOT:PSS. Similar to the first series post-treatment, the *PF* was nearly independent on the IL concentration, resulting in an average *PF* of 235 μW/K^2^m for BMIM-TFSI-SFS-F-PEDOT:PSS films. These values are among the highest *PFs* reported in the literature (Table S2) (Bubnova et al., 2011; Lee et al., 2014a; Park et al., 2014b; Wang et al., 2015; Yi et al., 2015; Fan et al., 2017). This enhancement in the *PF* is mainly due to the interaction of PSS^−^ groups with BMIM^+^, which tunes *n* in the PEDOT domains and thus significantly enlarges the *S* but does not compromise the σ too much. Besides, control samples were also prepared by treating the F-PEDOT:PSS and SFS-F-PEDOT:PSS films with pure methanol (i.e., MeOH-F-PEDOT:PSS and MeOH-SFS-F-PEDOT:PSS) to verify that the observed changes are not related to interactions of the solvent with the PEDOT:PSS film. MeOH-F-PEDOT:PSS and MeOH-SFS-F-PEDOT:PSS control films showed *PF*s of 92.5 and 189 μW/K^2^m, respectively. These values are smaller than those of the IL-F-PEDOT:PSS and IL-SFS-F-PEDOT:PSS films. Therefore, ILs in methanol has a more significant influence on the *S* and σ of PEDOT:PSS films than pure methanol.

### Mechanism of TE Properties Enhancement of ILs Treated Films

**Figure 2:**
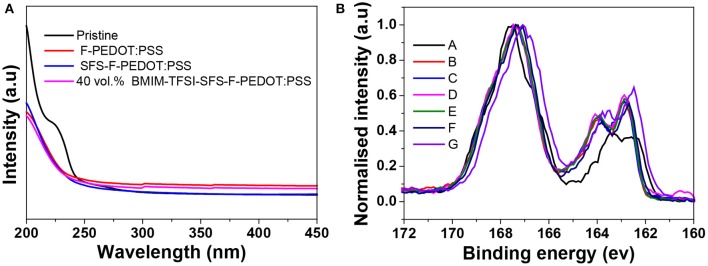
**(A)** UV-vis absorption spectra of the pristine, F-PEDOT:PSS, SFS-F-PEDOT:PSS and BMIM-TFSI-SFS-F-PEDOT:PSS films. **(B)** S 2p core-level spectra of the pristine, F-PEDOT:PSS, and BMIM-TFSI-SFS-F-PEDOT:PSS films treated with various vol.% BMIM-TFSI in methanol. Pristine (A), F-PEDOT:PSS (B), SFS-F-PEDOT:PSS (C), 40 vol.% BMIM-TFSI-SFS-F-PEDOT:PSS (D), 60 vol.% BMIM-TFSI-SFS-F-PEDOT:PSS (E), 80 vol.% BMIM-TFSI-SFS-F-PEDOT:PSS (F), and 100 vol.% BMIM-TFSI-SFS-F-PEDOT:PSS (G).

Figure 2A shows the UV absorption spectra of the pristine, F-PEDOT:PSS and SFS-F- PEDOT:PSS and BMIM-TFSI-SFS-F-PEDOT:PSS films. The absorption band located at 225 nm was assigned to the PSS. Generally, the decreased intensity of the absorption band at 225 nm of PEDOT:PSS films shows the loss of PSSH from the PEDOT:PSS films. Compared with the spectrum of F-PEDOT:PSS that showed remarkable reduction in intensity at 225 nm, both spectra of SFS-F-PEDOT:PSS and BMIM-TFSI-SFS-F-PEDOT:PSS were almost the same, indicating that, similar to other treatments (Xia et al., 2012), formamide-treatment effectively removed PSSH, resulting in an increase in the σ compared to the pristine film.

The S_2_p X-ray photoemission spectroscopy (XPS) was employed to study the influence of IL treatment (Figure 2B). The doublet XPS bands with binding energies between 166 and 172 eV were assigned to the S_2_p band of the sulfur atoms in PSS, whereas XPS peaks with binding energies between 162 and 166 eV were assigned to the S_2_p band of the sulfur atoms of PEDOT (Crispin et al., 2003; Kim et al., 2011). It can be seen from Figure 2B that the S_2_p intensity of PEDOT relative to PSS increases due to the removal of PSS after BMIM-TFSI treatment (Xia and Ouyang, 2009). The ratios of the PSS peak to PEDOT peak dropped from 2.5 for the pristine film to 1.02 for the SFS-F-PEDOT:PSS film, indicating that a substantial amount of PSS was removed in the treated films. The removal of PSS was also verified by the reduction in the film thickness. The thickness of the pristine film was 6 μm. In contrast, the thickness of IL-SFS-F-PEDOT:PSS, F-PEDOT:PSS and SFS-F-PEDOT:PSS film was reduced significantly to 2.3, 2.5, and 2.1 μm, respectively. Also, the S2p bands in the PEDOT increased and shifted to a higher binding energy (163.5 ev vs. 164.2 ev) after IL treatment, indicating the decrease in the doping level after treatment, and the lower doping level in CPs invariably led to a reduced σ. The dedoping with IL slightly affected the σ while significantly improve the *S*. The *PF* of PEDOT:PSS film attained its optimum value at a specific oxidation state as the *S* increased and the σ tends to decrease at lower oxidation levels, which is consistent with the previous report (Khan et al., 2000).

**Figure 3:**
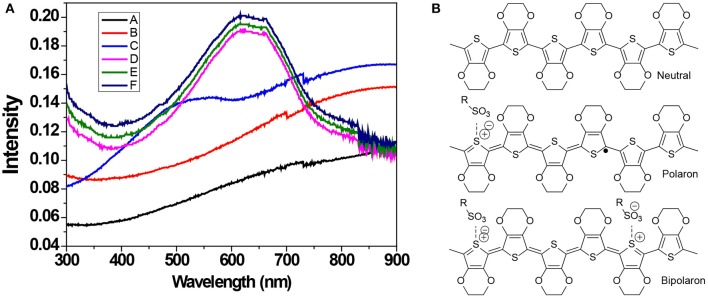
**(A)** UV-Vis-NIR absorption spectra of pristine, F-PEDOT:PSS, and BMIM-TFSI-SFS-F-PEDOT:PSS films treated with various vol.% of BMIM-TFSI in methanol. Pristine (A), F-PEDOT:PSS (B), SFS-F-PEDOT:PSS (C), 40 vol.% BMIM-TFSI-SFS-F-PEDOT:PSS (D), 60 vol.% BMIM-TFSI-SFS-F-PEDOT:PSS (E), and 80 vol.% BMIM-TFSI-SFS-F-PEDOT:PSS (F). **(B)** A schematic Illustration of the transition of PEDOT chains from bipolaron (a di-cation charge carrier) to polaron (a radical cation charge carrier) and neutral chain during dedoping with IL.

The oxidation level of PEDOT:PSS thin films treated with the chemical dedoping agent was determined with UV-Vis-NIR absorption spectroscopy (Figure 3A). PEDOT exists in a form of neutral, polaron (a radical cation charge carrier) and bipolaron (a di-cation charge carrier) state (Figure 3B). The pristine and F-PEDOT:PSS films show a broad absorption band covering the beginning of the infrared region domain, and this band is attributed to bipolaron (PEDOT^2+^). After binary dedoping (IL-F-PEDOT:PSS & IL-SFS-F-PEDOT:PSS), the oxidation level changes and the main peaks shifted to 900 nm for polaron (PEDOT^+^) and to 600 nm for neutral (PEDOT) redox states (Chung et al., 1984; Garreau et al., 2001; Im and Gleason, 2007; Bubnova et al., 2011). Nevertheless, this further binary dedoping with ILs steps leads to the formation of neutral states of PEDOT chains at a high intensity (i.e., the absorption intensity is higher for IL-SFS-F-PEDOT:PSS films than that for SFS-F-PEDOT:PSS films). This indicates that the PEDOT chains in the neutral state can remain in the neutral states upon binary dedoping but with a higher intensity, resulting in further enhancement in the *S* due to a lower *n*.

**Figure 4:**
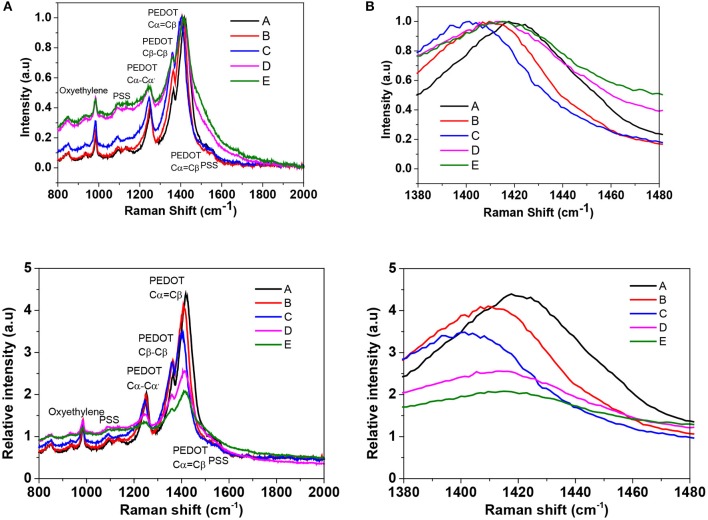
**(A)** Raman spectra of pristine PEDOT:PSS film and BMIM-TFSI-SFS-F-PEDOT:PSS films treated with different vol.% of BMIM-TFSI in methanol. Pristine (A), SFS-F-PEDOT:PSS (B), 40 Vol.% BMIM-TFSI-SFS-F-PEDOT:PSS (C), 60 Vol.% BMIM-TFSI-SFS-F-PEDOT:PSS (D), and 80 Vol.% BMIM-TFSI-SFS-F-PEDOT:PSS (E). **(B)** Zoom in spectra in the wavelength range of 1,380 to 1,480 cm^−1^.

Raman spectroscopy was used to further investigate the conformational changes in polymers and to study the change in the doping level of PEDOT:PSS films. Figure 4A displays the Raman spectra of the untreated and BMIM-TFSI-SFS-F-PEDOT:PSS treated films. The peaks at 987, 1,130, and 1,257 cm^−1^ came from the deformation of oxyethylene ring, the PSS component, and the vibrational mode of Cα-Cα' symmetric interring stretching, respectively (Garreau et al., 1999, 2001; Han et al., 2011; Farah et al., 2012). Also, the peak around 1,509 cm^−1^ was originated from the asymmetrical vibration of C_α_ = C_β_ in PEDOT and the peak around 1,369 cm^−1^ was assigned to the symmetric Cβ-Cβ stretching (Garreau et al., 1999). The structure of the pristine PEDOT is made up of the benzoid and quinoid forms in which the conjugated benzoid structure owns a localized π-electron largely unaffected by external stimuli, while the quinoid form of PEDOT holds a delocalized state of π-electrons which can be affected by solvent treatment (Ouyang et al., 2005). In the electrically active and oxidized state, there are positive charges on the PEDOT polymer backbone which are balanced with an anion, either a small molecular anion or a macromolecular anion such as the PSS^−^ (Ouyang et al., 2005). The vibrational bands at 1424 cm^−1^ can be ascribed to the stretching vibration on the Cα = Cβ of the five-member ring of the pristine PEDOT films. These vibrational bands were shifted to around 1,417 cm^−1^ for IL-SFS-F-PEDOT:PSS films (Figure 4B), suggesting a change from a predominately coil conformation (benzoid structure) to a mixed linear-coil conformation (quinoid structure) in the PEDOT chain (Garreau et al., 1999; Łapkowski and Pron, 2000), resulting in a quinoid dominant structure. The PSS chains are connected to the PEDOT chains through Coulombic interactions and have a coiled structure (core-shell) because of the repulsion among the long PSS chains (Lang et al., 2009a). This IL treatment could weaken the ionic interaction between the PEDOT and the PSS, resulting in phase separation between the PSS and the PEDOT and a linear conformation of the PEDOT chains. The same observations were reported on Raman analysis of EG treated PEDOT:PSS films (Ouyang et al., 2004). The partial removal of PSS is manifested by the reduction in the intensity of the Raman fingerprints of treated films compared to pristine films. Moreover, the peak at 1,424 cm^−1^ for the pristine shifted to 1,417 cm^−1^ upon IL treatment (Figure 4B), indicating that the doping level changed from bipolaron in the pristine to a neutral state in the IL-treated films as evident by UV-vis-NIR spectra and XPS. This Raman spectra along with UV-vis-NIR spectra and XPS indicate that the oxidation level changes from bipolaron to neutral upon dedoping lead to the slight decrease in the σ and the significant increase in the *S* due to the decrease in the *n* (Luo et al., 2013).

**Figure 5:**
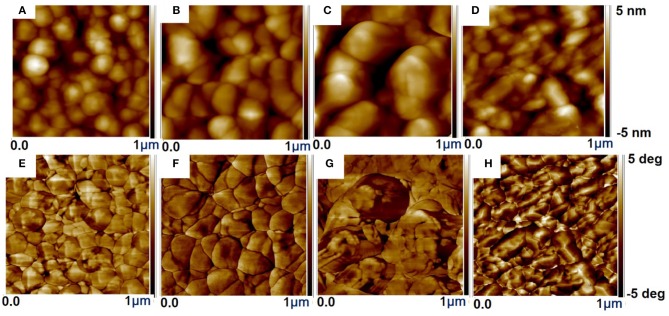
The AFM surface morphology IL-SFS-F-PEDOT:PSS films treated with various vol.% ILs in methanol. Height images: IL-SFS-F-PEDOT:PSS films treated with **(A)** 40 vol.% BMIM-TFSI, **(B)** 60 vol.% BMIM-TFSI, **(C)** 40 vol.% BMIM-OTf, and **(D)** 60 vol.% BMIM-BF_4_ in methanol. Phase images: IL-SFS-F-PEDOT:PSS films treated with **(E)** 40 vol.% BMIM-TFSI, **(F)** 60 vol.% BMIM-TFSI, **(G)** 40 vol.% BMIM-OTf, and **(H)** 60 vol.% BMIM-BF_4_ in methanol. The scanned area is 1 × 1 μm^2^ for each image.

The surface of treated films is highly non-uniform and with enhanced particle size, which leads to a more readily charge transport and thus an improved σ compared to the pristine film. While the untreated film does not show any apparent grains (Figures S2a,d), implying that the PSS chains are well-intermixed with the PEDOT chains and the PSS-rich domains mostly cover the film. The strong phase separation between the PSS-rich shell and the PEDOT-rich core besides the depletion of PSS chain were found in the treated films resulting in the interconnected large grains of PEDOT (Figure S2 and Figure 5) (Na et al., 2009; Luo et al., 2013, 2014). After dedoping with ILs the interconnection of the PEDOT-rich grains was enhanced, resulting in an enhanced σ compared to the pristine film. This could partially address why ILs treatment improves *PF* with a slight degradation of the σ.

Hall measurements were carried out to measure the *n* and the μ in order to further confirm the conformational change of the PEDOT chain and the phase segregation of the PSSH due to the binary dedoping with the IL. The results showed that all the prepared films were hole-type carrier dominated. In the *p*-type semiconductors, the σ is given by the relationship: σ = *en*μ, where *n, e*, and μ is the charge carrier concentration, electron charge, and charge carrier mobility, respectively (Hiroshige et al., 2007). The σ of the pristine and treated PEDOT:PSS films (as shown in Table 2) is in the same order of magnitude with the measured value (Table S1), demonstrating the reliability of the current measurements. The slightly decreased σ of the ILs dedoped PEDOT:PSS film was mainly caused by the one-order-of-magnitude reduction in the *n* as the μ varied slightly. This may be ascribed to the interaction between ILs molecules and the PSS monomers, and thus inhibit the carrier supply from the PSS. Generally, the *S* effectively varies with the slight doping or dedoping concentration as manifested in the current work that the dedoping effect of ILs contributes to the significant enhancement in the *S*.

**Table 2:** Calculated σ and experimentally determined *n* and μ of the sequential formamide (three times) and 100 mM SFS pre-treated PEDOT:PSS films at various vol.% BMIM-TFSI in methanol by the aid of blowing N_2_ gas.

**Treatment methods (vol.% BMIM-TFSI in methanol)**	**μ (cm^**2**^/Vs)**	***n* (cm^**−3**^)**	**σ (S/cm)**
Untreated	0.42 ± 0.03	4.1 ± 0.3 × 1018	0.28 ± 0.1
F-PEDOT:PSS	1.08 ± 0.1	1.56 ± 0.1 × 1022	2693.7 ± 201.8
0	0.58 ± 0.04	5.32 ± 0.5 × 1021	493.72 ± 41
20	0.51 ± 0.05	5.11 ± 0.3 × 1021	416.97 ± 37
40	0.48 ± 0.04	5.01 ± 0.7 × 1021	384.77 ± 31
60	0.43 ± 0.04	4.87 ± 0.63 × 1021	335.05 ± 29
80	0.41 ± 0.05	4.65 ± 0.45 × 1021	305.04 ± 21
100	0.38 ± 0.05	4.61 ± 0.39 × 1021	280.2 ± 16

**Figure 6:**
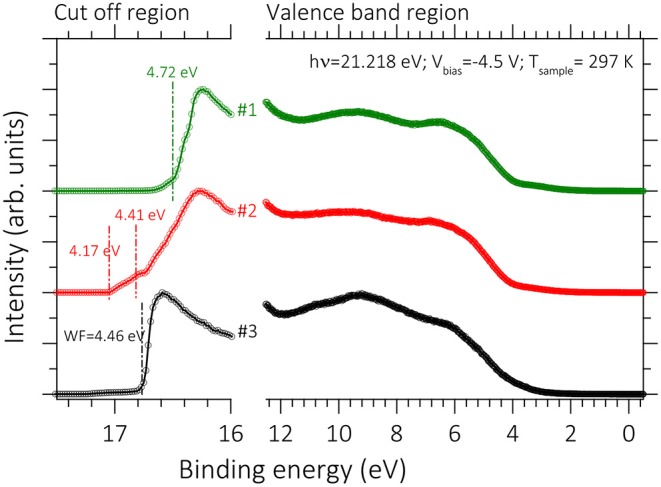
UPS spectra of pristine and BMIM-TFSI-SFS-F-PEDOT:PSS films treated with various vol.% BMIM-TFSI in methanol using He I photon (21.22 eV). Pristine (#1), BMIM-TFSI-SFS-F-PEDOT:PSS treated with 0 vol.% (#2), and 40 vol.% BMIM-TFSI in methanol. Φ values calculated from the cut off position (estimated by linear extrapolation).

In addition to TE performance of the films, the ILs treatment on the PEDOT:PSS films may also affect other properties of PEDOT:PSS films relevant to the device operation, such as the work function (Φ). Ultraviolet photoelectron spectroscopy (UPS) is a key technique to determine the Φ of surfaces by measuring the secondary-electron cut-off (Ec). The influence of the Φ and the valence band on the pristine and treated PEDOT:PSS films was determined using UPS measurements (Figure 6). The Φ could be obtained from the equation, Φ = *hv -Ec*, based on the UPS measurements where the spectral width secondary-electron cut-off (*Ec*) is obtained from the energy gap between the inelastic secondary electron emission cutoff and the Fermi edge and *hv* is the photon energy of the UPS light source (Janardhanam et al., 2015; Kim et al., 2018). We found that IL treatment caused a reduction in the Φ from 4.7 to 4.4 eV (Figure 6). For PEDOT:PSS thin films, a range of the Φ from 4.7 to 5.4 eV has been reported (Scott et al., 1999; Greczynski et al., 2001; Mäkinen et al., 2001; Havare et al., 2012) which is similar to the result shown in Figure 6. The spread in the Φ values is assumed to be related to differences in the top layer, which may contain an excess of the PSS (Jönsson et al., 2003; Huang et al., 2005; Snaith et al., 2005; Crispin et al., 2006). The PSS-rich top layer may be modified by the addition of high-boiling solvents (Huang et al., 2005; Snaith et al., 2005; Hwang et al., 2006) and other processing conditions (Koch et al., 2007).

The *S* gives the energetic difference between the transport level (*E*μ*)* and the Fermi level (*E*_*F*_) by its value and the transport type (*p* or *n* by its sign). The theoretical result indicates, the *S* generally depends on the Fermi level as expressed (Fritzsche, 1971; Nollau et al., 2000):

(1) $$\begin{array}{l} S\left(T\right)=\frac{1}{eT}\frac{\int \left[E_{F}\left(T\right)-E\right]\delta \sigma \left(E\right)dE}{\int \delta \sigma \left(E\right)dE} \end{array}$$

where δσ*(E)* is the differential conductivity at energy *E, E*_*F*_ is the Fermi level, and *e* is the electronic charge. The integrations extend over the entire energy range. This derivation holds for both delocalized and localized states, i.e., band and hopping transport, only the assumption of a Fermi system is necessary. With a further assumption of unipolar charge carrier transport at one narrow transport level (*E*μ) Equation (1) provides:

(2) $$\begin{array}{l} S\left(T\right)=\frac{E_{F}\left(T\right)-E_{\mu }}{eT} \end{array}$$

In the transport state, the energy difference *E*_*F*_*(T)* – *E*_μ_ is interrelated to the carrier concentration *n(T)* (Nollau et al., 2000; Sze and Ng, 2006). The variation of about one order decrease of the carrier concentration will increase the *E*_*F*_*(T)* – *E*_μ_, and thus enhance the *S* according to Equation (2). A similar tendency was observed in doped organic semiconductors between the *n* and TE properties including the σ and *S* (Nollau et al., 2000). Moreover, as the *S* relies on the local band structure of the material, the removal of the PSS could transform the local band structure of PEDOT:PSS. Thus, the enhancement in the *S* may be due to a probable change in the band structure.

**Figure 7:**
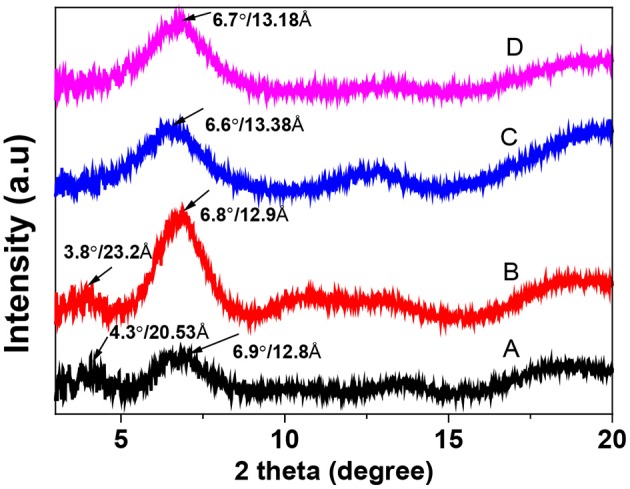
X-ray diffraction (XRD) patterns of pristine and BMIM-TFSI-SFS-F-PEDOT:PSS films treated at various vol.% BMIM-TFSI in methanol. A refers to Pristine, B refers to F-PEDOT:PSS, C refers to SFS-F-PEDOT:PSS, and D refers to 40 vol.% BMIM-TFSI-SFS-F-PEDOT:PSS.

The crystallinity of PEDOT:PSS films was studied with the XRD (Figure 7). The pristine film displayed two characteristic peaks at *2*θ values of 4.3° and 6.9° corresponding to the lattice *d* spacing of 20.5 Å and 12.8 Å, which were calculated in terms of Bragg's law (*2dsin* θ = λ). They can be assigned to the lamella stacking distance *d* (100) of the two distinct alternate ordering of PEDOT and the PSS chain. BMIM-TFSI-SFS-F-PEDOT:PSS films showed a slight change from 12.8 to 13.8 Å in the lamella stacking distance of the alternate ordering of PEDOT and the PSS chain (Figure 7). It also displayed relatively sharper diffraction peaks with the higher intensity in the low angle reflections at *2*θ of 6.7° in comparison with the XRD patterns of the pristine PEDOT:PSS. This corresponded separately to the lamella stacking distance *d* (100) of two distinct alternate orderings of the PEDOT and the PSS chains, suggesting a higher crystallization degree of the PEDOT:PSS film. Besides, for BMIM-TFSI-SFS-F-PEDOT:PSS film, the *2*θ is further shifted to 6.7° corresponding to a lattice *d* spacing of 13.8 Å, and a fair improvement in diffraction peak intensity was observed. These results demonstrate that the IL treatment makes PEDOT:PSS films to prefer a specific lamella stacking between the PEDOT chains, resulting in the improved crystallinity of the PEDOT:PSS films. The (100) diffraction peak intensity was significantly improved, attributable to the improvement of the number of ordered aggregates associated with interchain π-π stacking between the PEDOT chain and the enhancement of the crystallinity of the PEDOT:PSS film. Therefore, the XRD results displayed that compared to the pristine films, IL dedoped PEDOT:PSS films showed an enhanced interchain coupling of PEDOT:PSS with a more densely packed PEDOT and lamella stacking between two assemblies, resulting in an improved *S* of the films through interface scattering (Kim et al., 2014; Wang et al., 2018).

Moreover, the thermal properties of PEDOT:PSS films were investigated and the ρ, *b, Cp*, and κ of the pristine and BMIM-TFSI-SFS-F-PEDOT:PSS films are measured (Table 3). The κ of the pristine PEDOT:PSS film was in accordance with those reported by independent groups (Lee et al., 2014a; Wang et al., 2015, 2018). The pulsed light heating thermoreflectance method was used to determine *b* at room temperature (Baba et al., 2011; Kyaw et al., 2018). The thermalreflectacne signals of the pristine and 40 vol.% BMIM-TFSI-SFS-F-PEDOT:PSS film after the nanosecond-pulse heating was detected by the probe beam (Figure S3). The effusivity values derived from the curve fitting are given in Table 3. The *Cp* was obtained using DSC and the ρ_*m*_ was calculated from the mass and the volume of the film at room temperature. As illustrated in Table 3 the κ of BMIM-TFSI-SFS-F-PEDOT:PSS films was reduced compared to that of the pristine PEDOT:PSS film. The reduced κ could be because of the removal of excess PSS. Therefore, the dimensionless *ZT* at 300K was calculated based on the obtained cross plane κ of 0.27 W/mK and the corresponding highest *PF* of 239.2 μW/K^2^m. The *ZT* value of the BMIM-TFSI-SFS-F-PEDOT:PSS film was ~ 0.26 whereas the pristine PEDOT:PSS was ~5.6 × 10^−6^ at 300K. This dramatic enhancement in *ZT* in the 40 vol.% BMIM-TFSI-SFS-F-PEDOT:PSS films indicate that our treatment technique is effective for the enhancement of the TE properties of PEDOT:PSS film.

**Table 3:** The thermal properties (ρ, *Cp, b*, and κ) of the pristine and 40 vol.% BMIM-TFSI-SFS-F-PEDOT:PSS films. The obtained κ values were at room temperature.

**Code**	***b* (J/S^**0.5**^m^**2**^)**	***Cp* (J/gK)**	***ρm* (g/cm^**3**^)**	**κ (W/m)**
Untreated	1175	1.5	1.6	0.57
40 vol.% BMIM-TFSI-SFS-F-PEDOT:PSS	785.2	1.56	1.62	0.27

### PEDOT:PSS Film Stability Study

**Figure 8:**
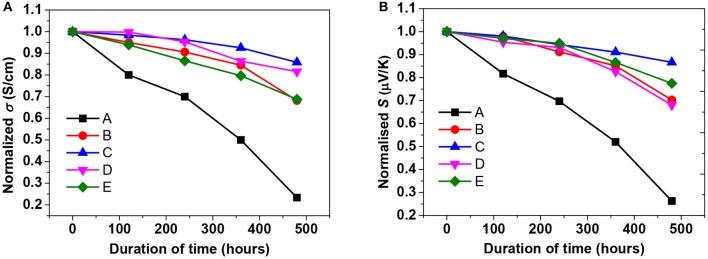
The reduced TE properties of the pristine, formamide treated, and BMIM-TFSI-SFS-F-PEDOT:PSS films treated with various vol.% BMIM-TFSI in methanol. **(A)** σ and **(B)** *S* of the pristine and treated PEDOT:PSS films. Pristine (A), F-PEDOT:PSS (B), SFS-F-PEDOT:PSS (C), 40 vol.% BMIM-TFSI-SFS-F-PEDOT:PSS (D), and 60 Vol.% BMIM-TFSI-SFS-F-PEDOT:PSS (E). Vertical: *Si/S0* and σ*i/*σ*0* where (σ*0* and *S0*) and (*Si* and σ*i*) are *S* and σ at t = 0 h and the time at which the *S* and σ were recorded respectively, kept over 480 h under high humidity (75% RH) and high temperature (70°C) conditions.

The PEDOT:PSS film stability by measuring the σ, κ, and *S* of their films at a given humidity and temperature was investigated. The pristine and treated films were put in a humidity chamber at 75% RH and 70°C for up to 480 h to investigate the PEDOT:PSS film stability. In this test, 20 batches of samples were prepared and the σ, κ, and *S* were periodically monitored throughout the stability test. The results of the pristine PEDOT:PSS film were in agreement with the previous reports by the majority of groups on atmospheric exposure of PEDOT:PSS films (Nardes et al., 2008; Kim et al., 2011; Alemu et al., 2012; McCarthy et al., 2014; Cho et al., 2016). As shown in Figure 8, the σ and *S* of all samples gradually decreased with the increasing exposure time. The relative decrements in the σ in the harsh conditions after 480 h for the pristine PEDOT:PSS film was 77%, while ILs treated PEDOT:PSS films reduced by ~22%, indicating the long-term stability was enhanced through the ILs treatment. It was well noted that the significant reduction in the σ of pristine PEDOT:PSS film was owing to the hygroscopic and acidic PSS that picks up water easily (De Jong et al., 2000; Fehse et al., 2008). Since the σ deterioration occurred due to absorption of water in the PSS phase (Van Reenen and Kemerink, 2014), the reduction of the PSS in treated PEDOT:PSS film resulted in less water absorption, and subsequently the film was more stable even in a harsh environment. Furthermore, the highly compact-structure brought by the depletion of PSS and the polymer rearrangement may improve the σ as well as stability (Nardes et al., 2008). In addition, the stability enhancement may relate to a strong ionic interaction between sulfonate anions and bulky imidazolium cations that blocking the penetration of water into the PEDOT:PSS film and reducing the water uptake. Blending imidazolium derivatives with acidic PEDOT:PSS solutions leads to neutralization of the solution, remarkably enhancing the stability with minimal loss of the σ; in well agreement with the previous report (Cho et al., 2016).

Furthermore, the cross plane κ was observed to be slightly increased for 40 vol.% BMIM-TFSI-SFS-F-PEDOT:PSS films while the cross-plane κ was noticed to be decreased for the pristine PEDOT:PSS film with harsh conditions. The result is in good accordance with a previously observed decline in the elastic modulus of PEDOT:PSS at high humidity conditions (Lang et al., 2009b), i.e., κ = (elastic constant)^1/2^ (Hsieh et al., 2011). Hence, the slight increase of κ in BMIM-TFSI-SFS-F-PEDOT:PSS films could be due to the film having less PSS and hence less softening conditions and less hygroscopic.

**Figure 9:**
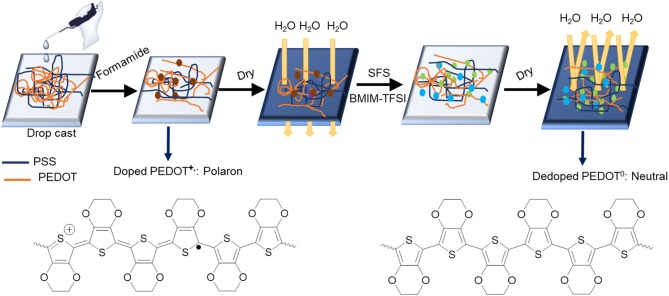
Schematic illustrations of a model for the mechanism of TE properties enhancement for ILs-SFS-F-PEDOT:PSS.

**Figure 10:**
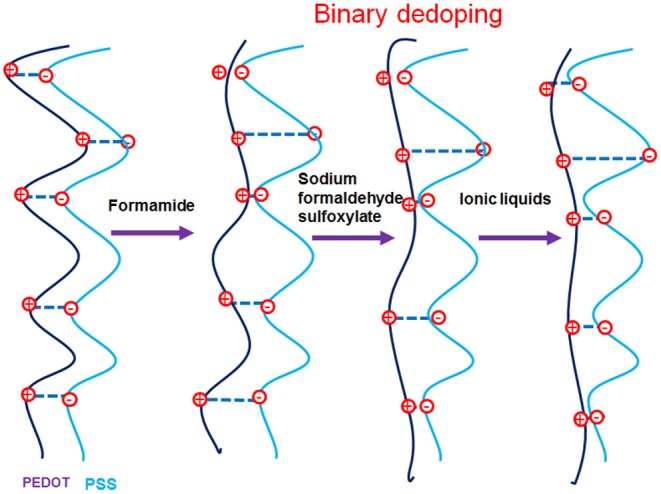
Illustrations of conformational changes of PEDOT chains with binary dedoping with SFS and ILs.

Based on our various observations, we suggest a model for the mechanism of TE properties enhancement for ILs-SFS-F-PEDOT:PSS films (Figure 9). Figure 10 also illustrates conformational changes of PEDOT chains for untreated and treated films. Formamide, a polar solvent, with a high dielectric constant induces a strong screening effect between the counter ions and the charge carriers, reducing the interactions between the negatively charged PSS and the positively charged PEDOT. This results in an enlargement of PEDOT chains and the easier removal of PSS due to the change in their conformation from coils to elongated structures (Kim et al., 2002). When SFS (HOCH_2_SO_2_Na) was further introduced into the PEDOT:PSS films, HOCH_2_${\text{SO}}_{2}^{-}$ and Na^+^ ions penetrated into the PEDOT:PSS film, the HOCH_2_${\text{SO}}_{2}^{-}$ ions could replace the PSS counter ion and bind to the PEDOT segment as the new counter ion, and the Na^+^ ions bound to the PSS anions during the treatment. The decreased steric hindrance and dramatically reduced binding effect exerted by PSS led to the conductive PEDOT chain further to elongate and achieve an extended conformation. This extended conformation led to stronger interchain interactions, resulting in a significantly enhanced σ of PEDOT:PSS that facilitates charge transport among the polymer chains (Xia and Ouyang, 2011; Culebras et al., 2014). Further addition of ILs e.g. BMIM-TFSI effectively segregated the PEDOT from the PSS, resulting in conformational changes and in turn an increase in mobility. Also, BMIM-TFSI treatment led to electrostatic interactions of the negatively charged PSS with the positively charged BMIM cation and dissociation of the ionic bond between BMIM and TFSI. This led to further elongation of the conductive PEDOT chain to achieve an extended conformation. Figure 10 illustrates conformational changes of the PEDOT chains and a sequential formamide and binary dedoping. This linear-like conformation could change chemical states of PEDOT chains that affect the carrier density in the PEDOT:PSS films (Park et al., 2014b).

## Conclusion

In this study, two series post-treatments using ILs as one of the key steps were reported. Both series post-treatments considerably enhanced TE properties of PEDOT:PSS films. The first series post-treatment resulted in a big jump in the power factor from 63.6 to 137.8 μW/K^2^m, corresponding to post-treatment with formamide only, and post-treatment with both formamide and IL, respectively. An additional post-treatment step with SFS was introduced before applying to IL treatment, and the power factor could be further improved to more than 230 μW/K^2^m. A large tendency to enhance the power factor likely mainly originated from the improvement in the Seebeck coefficient *S*, which was increased from 14.9 to 28.5 and then 61 μV/K although the electrical conductivity σ reduced from a few thousand to in the range of 630–650 S/cm. Compared to the F-PEDOT:PSS film, the improvement in the *S* is because of the decrease in the carrier concentration *n* by roughly 70% in the case of BMIM-TFSI treated PEDOT:PSS. The enhancement in the *S* could also be explained by the dedoping process as evidenced by the absorption spectra in which a characteristic absorption peak at 600 nm appeared. The types of anions associated with ILs also played a role in affecting the magnitude of the power factor in an order of TFSI > OTf >BF_4_. On the other hand, the cross-plane κ reduced from 0.57 W/mK for the pristine film to 0.27 W/mK for BMIM-TFSI-SFS-F-PEDOT:PSS film is largely due to the removal of the PSS. Hence, under the optimum treatment condition, the estimated *ZT* of ~0.26 was achieved at 300 K, revealing the potential in the application for harvesting low-grade heat or waste thermal energy.

## Data Availability Statement

All datasets generated for this study are included in the article/Supplementary Material.

## Author Contributions

TY, WC, and JX conceived and designed the experiments. TY and YZ performed the experiments and contributed to the film fabrication, measurement, and data analysis. AK, XW, and JS contributed to analysis tools, helped with the analysis, and discussed the data. TY wrote the paper. WC and JX helped revise the paper.

### Conflict of Interest

The authors declare that the research was conducted in the absence of any commercial or financial relationships that could be construed as a potential conflict of interest.
