# Supplementary material for: Improved Thermoelectric Properties and Environmental Stability of Conducting PEDOT:PSS Films Post-treated With Imidazolium Ionic Liquids
Source: Front Chem. 2020 Jan 8;7:870. doi: 10.3389/fchem.2019.00870 (PMC6960405; doi:10.3389/fchem.2019.00870)
Supplement: Supplementary file 1 [file Table_1.pdf]

Supplementary Material

Improved thermoelectric properties and environmental stability of conducting PEDOT:PSS films post-treated with imidazolium ionic liquids

Temesgen Atnafu Yemata[1,2], Yun Zheng [1], Aung Ko Ko Kyaw[1,3], Xizu Wang[1], Jing Song [1], Wee Shong Chin[2*], and Jianwei Xu[1,2*]

[1]Institute of Materials Research and Engineering; Agency for Science, Technology, and Research (A*STAR), 2 Fusionopolis Way, Singapore 138634, Republic of Singapore

[2]Department of Chemistry, National University of Singapore, 3 Science Drive 3, Singapore 117543, Republic of Singapore

[3]Department of Electrical and Electronic Engineering, Southern University of Science and Technology, Shenzhen 518055, P.R. China

*** Correspondence:**

jw-xu@imre.a-star.edu.sg (J. Xu) and chmcws@nus.edu.sg (W. S. Chin)

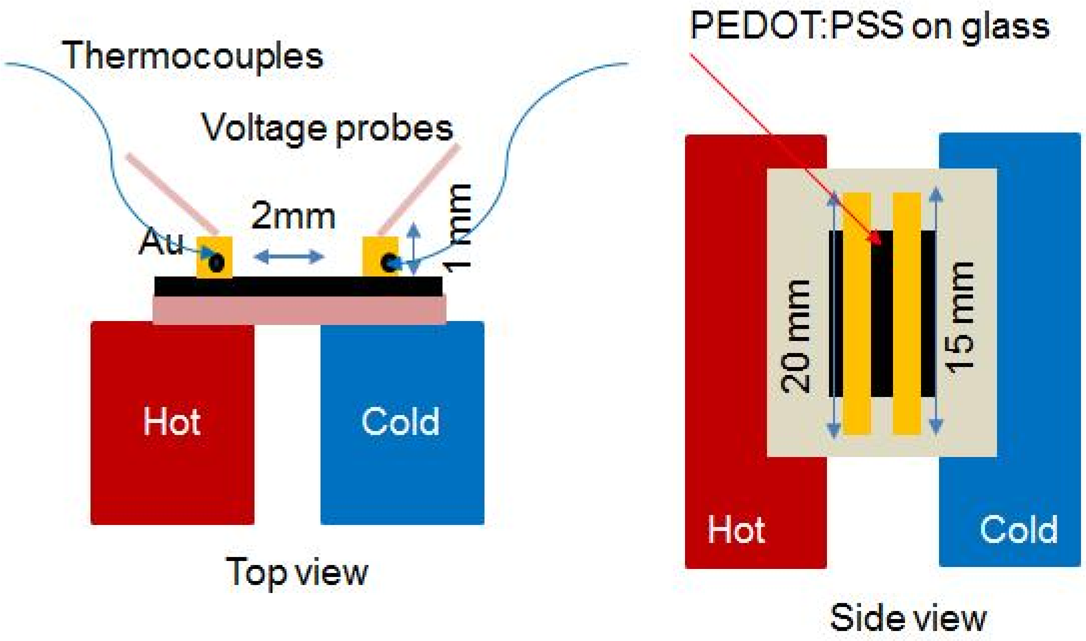

Figure S1. TE characterization of the PEDOT:PSS films. Schematic illustration of the *S* measurement setup and detailed electrode geometry.

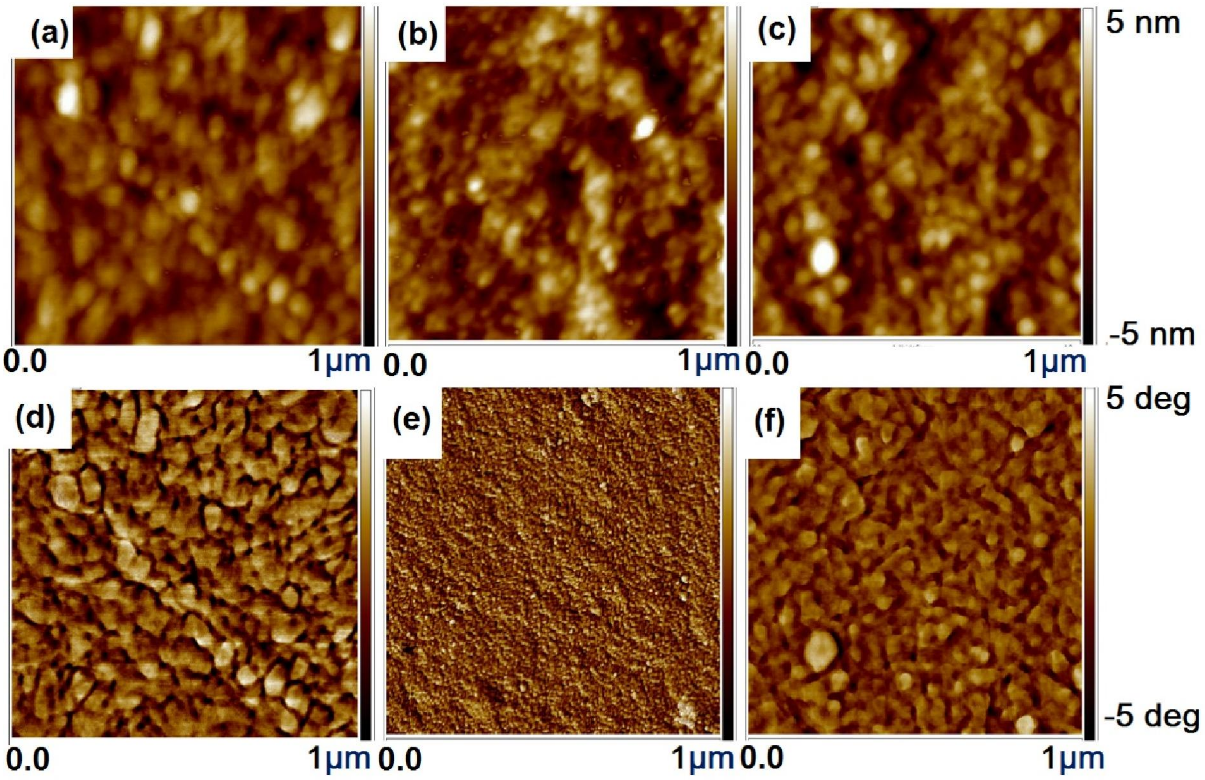

Figure S2. The AFM surface morphology of pristine and SFS-F-PEDOT:PSS films at various concentration SFS in water. Height images: (**a**) pristine, (**b**) 0, and (**c**) 100 mM SFS in water. Phase images: (**d**) pristine, (**e**) 0, and (**f**) 100 mM SFS in water. The area scanned is 1×1 μm^2 for each image.

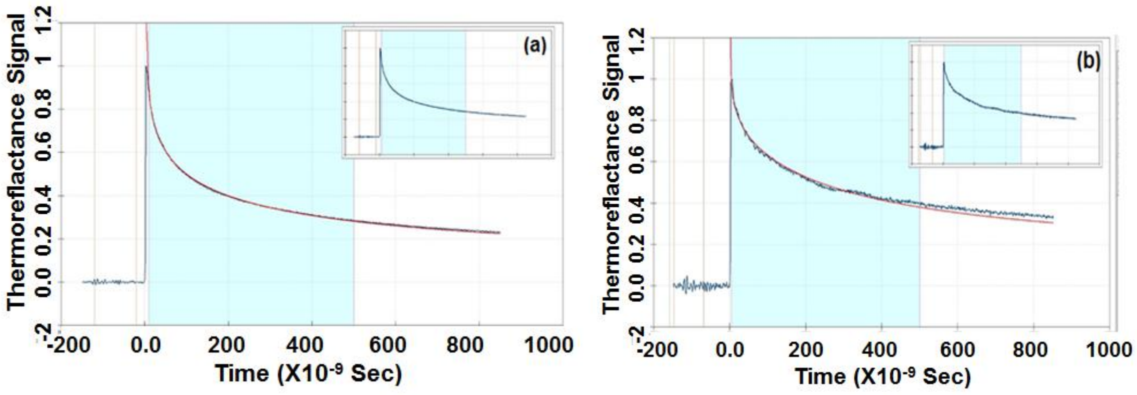

Figure S3. The normalized thermal reflectance signals of pristine and BMIM-TFSI-SFS-F-PEDOT:PSS films coated with 100 nm-thick-Al after the nanosecond-pulse heating. (**a**) Pristine and (**b**) 40 vol.% BMIM-TFSI-SFS-F-PEDOT:PSS films. The *b* of the PEDOT:PSS

films are extracted from the fitted temperature response curve which is represented with the solid red lines.

Table S1. Thermoelectric properties (*σ*, *S* and *PF)* of the sequential formamide (three times) and 100 mM SFS pre-treated films at various vol.% BMIM-TFSI in methanol.

Vol.% BMIM-TFSI-SFS-F-PEDOT:PSS	*σ* (S/cm)	*S* (μV/K)	*PF* (μW/mK^2)
0	693.01 ± 7	51.78 ± 0.8	185.77 ± 4.3
20	645.9±6	60.44±0.5	235.9±4.3
40	641±5	61.09±0.7	239.2±4.5
60	635.1±7	60.7±0.6	234±5.5
80	633.2±5	60.81±0.8	234.1±5.1
100	631.3±4	60.56±0.6	231.5±4.5

Table S2. Comparison of this work with previous reports in the literature on some typical sequential treated PEDOT based TE materials

Ref.	Dedoping Treatment	*σ* (S/cm)	*S* (μV/K)	*PF* (μW/K^2m)	*k* (W/mK)	*ZT*
(Bubnova et al., 2011)	PEDOT:TOS dedoping with TDAE ($C_{10}H_{24}N_4$)	67	220	324	-	-
(Park et al., 2014)	PEDOT:PSS treated with a mixture of DMSO and hydrazine	578	67	112	-	
(Yi et al., 2015)	Addition of DMSO in to PEDOT:PSS and then treated with Poly(ethylene oxide)	1061	38.4	157.35	-	-
(Lee et al., 2014b)	PEDOT:PSS treated with DMSO (C_2H_6OS), then DMSO and hydrazine (N_2H_4)	677	41	115.48	0.17	0.2
(Wang et al., 2015)	PEDOT:PSS treated with DMSO/$NaBH_4$	~580	~40	98.1	0.451	0.064
(Lee et al., 2014a)	PEDOT:PSS treated with p-toluenesulfonic acid monohydrate ($C_7H_8O_3S \cdot H_2O$)	~1300	~50	318.4	0.3	0.31

	, then hydrazine (N_2H_4) /DMSO solution					
(Fan et al., 2017)	PEDOT:PSS treated with H_2SO_4, then NaOH	2170	39.2	334	-	-
This work	PEDOT:PSS treated with formamide ($HCONH_2$), then SFS (CH_3NaO_3S) and then 40 Vol.% BMIM-TFSI	641	61.1	239.2	0.27	0.26

References

Bubnova, O., Khan, Z.U., Malti, A., Braun, S., Fahlman, M., Berggren, M., and Crispin, X. (2011). Optimization of the thermoelectric figure of merit in the conducting polymer poly (3, 4-ethylenedioxythiophene). *Nat. Mater.* 10**,** 429.

Fan, Z., Li, P., Du, D., and Ouyang, J. (2017). Significantly Enhanced Thermoelectric Properties of PEDOT: PSS Films through Sequential Post‐Treatments with Common Acids and Bases. *Adv. Energy Mater.* 7**,** 1602116.

Lee, S.H., Park, H., Kim, S., Son, W., Cheong, I.W., and Kim, J.H. (2014a). Transparent and flexible organic semiconductor nanofilms with enhanced thermoelectric efficiency. *J. Mater. Chem. A* 2**,** 7288-7294.

Lee, S.H., Park, H., Son, W., Choi, H.H., and Kim, J.H. (2014b). Novel solution-processable, dedoped semiconductors for application in thermoelectric devices. *J. Mater. Chem. A* 2**,** 13380-13387.

Park, H., Lee, S.H., Kim, F.S., Choi, H.H., Cheong, I.W., and Kim, J.H. (2014). Enhanced thermoelectric properties of PEDOT: PSS nanofilms by a chemical dedoping process. *J. Mater. Chem. A* 2**,** 6532-6539.

Wang, J., Cai, K., and Shen, S. (2015). A facile chemical reduction approach for effectively tuning thermoelectric properties of PEDOT films. *Org. Electron.* 17**,** 151-158.

Yi, C., Wilhite, A., Zhang, L., Hu, R., Chuang, S.S., Zheng, J., and Gong, X. (2015). Enhanced thermoelectric properties of poly (3, 4-ethylenedioxythiophene): poly (styrenesulfonate) by binary secondary dopants. *ACS Appl. Mater. Interfaces* 7**,** 8984-8989.
